# The novel peptide LCGM-10 attenuates metabotropic glutamate receptor 5 activity and demonstrates behavioral effects in animal models

**DOI:** 10.3389/fnbeh.2024.1333258

**Published:** 2024-02-07

**Authors:** Anton V. Malyshev, Vsevolod V. Pavshintcev, Nikita A. Mitkin, Iuliia A. Sukhanova, Vasilina R. Gedzun, Alexander S. Zlobin, Igor I. Doronin, Gennady A. Babkin, Tomi K. Sawyer

**Affiliations:** ^1^Lactocore Inc., Newton, MA, United States; ^2^Maestro Therapeutics, Southborough, MA, United States

**Keywords:** group I metabotropic glutamate receptor, molecular docking, peptide drugs, novel treatment, drug screening, stimulant action

## Abstract

We employed a structural bioinformatics approach to develop novel peptides with predicted affinity to the binding site for negative allosteric modulators (NAMs) of metabotropic glutamate receptor 5 (mGluR5). Primary screening in zebrafish (*Danio rerio*) revealed a stimulatory effect of two peptides, LCGM-10 and LCGM-15. Target validation studies using calcium ion flux imaging and a luciferase reporter assay confirmed mGluR5 as the target. LCGM-10 showed greater potency than LCGM-15; it was comparable to that of the mGluR5 NAM 2-methyl-6-(phenylethynyl) pyridine (MPEP). Rodent behavioral screening in the open field and elevated plus maze revealed increased locomotor activity in both tests after acute LCGM-10 treatment, supported by further analysis of home cage spontaneous locomotor activity (SLA). The stimulating effect of a single LCGM-10 administration on SLA was evident up to 60 min after administration and was not accompanied by hypokinetic rebound observed for caffeine. According to our results, LCGM-10 has therapeutic potential to treat hypo- and dyskinesias of various etiologies. Further investigation of LCGM-10 effects in the delay discounting model of impulsive choice in rats revealed reduced trait impulsivity after single and chronic administrations, suggesting potential implication for attention deficit hyperactivity disorder, obsessive compulsive disorder, and addictions.

## 1 Introduction

The development of novel treatments normalizing metabotropic glutamate receptor 5 (mGluR5) function in the central nervous system (CNS) is of great importance. mGluR5 is expressed at high levels in several brain regions (Brain tissue expression of GRM5, n.d.) and is involved in a multitude of brain-related illnesses (Su et al., 2022) including fragile X syndrome (FXS) (Bear et al., 2004; Brašić et al., 2022), depression (Pilc et al., 2008; Esterlis et al., 2022), Parkinson’s disease (Pisani et al., 2003; Azam et al., 2022), Alzheimer’s disease (Sokol et al., 2011), attention deficit hyperactivity disorder (ADHD) (Elia et al., 2010), and addictions (Niedzielska-Andres et al., 2021). Because direct-acting agonists produce substantial adverse effects and eventually lead to profound receptor desensitization, the development of allosteric modulators has been at the forefront of G protein–coupled receptor (GPCR) drug development (Marino and Conn, 2006; Kampen et al., 2022). Negative allosteric modulators (NAMs) and positive allosteric modulators (PAMs) will only modulate receptor activity in the presence of the endogenous agonist, which is not possible with orthosteric ligands and enables more specific control of the tissue response (Wood et al., 2011; Stansley and Conn, 2019). The non-competitive mechanism of action of NAMs makes them relatively unaffected by high concentrations of glutamate that may be present in disease states (e.g., stroke, epilepsy, neuropathic pain, etc.) (Pin and Archer, 2002).

The development of new drugs with an allosteric mode of action has been greatly enhanced by advances in X-ray crystallography and cryo-electron microscopy, which have provided databases of high-resolution GPCR structures in complex with ligands and intracellular effectors for the docking studies (Congreve et al., 2020). Here we employed computational modeling to search for peptides that interact with the NAM site of mGluR5. We focused on peptides as potential therapeutics because of their safety and tolerability profiles, which are superior to those of other small molecules (Lau and Dunn, 2018; Malyshev et al., 2021; Mitkin et al., 2022). We identified four novel, previously undescribed peptides with predicted affinity to the mGluR5 NAM site based on *in silico* docking studies and revealed that two of them, LCGM-10 and LCGM-15, have a potential stimulating effect in zebrafish. mGluR5 modulation by LCGM-10 in a wide range of concentrations was supported by calcium ion flux imaging and a luciferase assay, using known mGluR5 agonists and antagonists. Several mGluR5 NAMs have been investigated for the treatment of drug addiction (Gass et al., 2009), FXS (Bear et al., 2004), Parkinson’s disease L-dopa-induced dyskinesia (Dekundy et al., 2006; Johnston et al., 2010; Grégoire et al., 2011), and depression (Palucha and Pilc, 2007), showing promising results in animal models. Several mGluR5 non-competitive antagonists have been tested for potential efficacy in clinical trials, including mavoglurant (FXS, cocaine use disorder, L-dopa induced dyskinesias, obsessive compulsive disorder [OCD], and Huntington’s disease), basimglurant (major depressive disorder), GET 73 (alcohol use disorder), and ADX10059 (gastroesophageal reflux disease, dental anxiety, and migraine) (Clinical Trials Register, n.d.; ClinicalTrials.gov, n.d.). The efficacy of mGluR5 antagonists has been reported in trials with patients with gastroesophageal reflux; however, data from patients with Parkinson’s disease or FXS have not been as robust as hoped (Witkin et al., 2022). Fenobam was approved for use as an anxiolytic prior to its recognition as a mGluR5 NAM (Porter et al., 2005).

In this study, *in vivo* behavioral characterization of the LCGM-10 and LCGM-15 peptides revealed hyperlocomotion in intact animals in the standard behavioral paradigms of open field (OF) and elevated plus maze (EPM), as well as in the home cage conditions. Additionally, in the delay discounting model of impulsivity, we found that single and chronic LCGM-10 treatment potently reduced impulsivity in rats. Our results suggest the need for additional studies of LCGM-10 as a potential treatment for hypo-and dyskinesias as well as for ADHD, OCD, and pathological conditions associated with impulsive behavior (drug addiction and gambling).

## 2 Materials and methods

### 2.1 Animals

Wildtype *D. rerio* (128 fish, shortfin phenotype, 6–8 months of age, male to female ratio 50: 50) were kept in a ZebTEC recirculating system (Tecniplast S.p.a, Buguggiate, Italy), and housed under a 14/10-h photoperiod (lights on at 08: 00 and off at 22: 00). The system parameters were maintained automatically with water set at 28°C, pH 6.8–7.5, 550–700 mOsm/L, and constant aeration. Feeding was carried out twice a day with special food for fish (Special Diet Services, Scientific Fish Food, SDS 300–400). In total, 30 male Sprague Dawley rats were used in the behavioral screening study, and 53 male Wistar rats in the locomotion study (*N* = 23) and the delay discounting test (*N* = 30). The animals were housed under constant environmental conditions (12-h photoperiod at 22 ± 2°C) with *ad libitum* access to food and water. All animal experiments were conducted in accordance with the European Directive 2010/63/EU of the European Parliament (Council of 22 September 2010 on the protection of animals used for scientific purposes) and were approved by the local Bioethics Commissions. All manipulations with animals were carried out at the end of the 14-day adaptation period.

### 2.2 Drug treatment and behavioral screening in *Danio rerio*

LCGM peptides (supplied by Lactocore Inc., synthesized by Peptide 2.0 Inc. [Herndon, VA, United States], 98% purity) were administered at a dose of 1 mg/kg (intraperitoneal, prepared in saline on the day of the experiment). The peptides had the following amino acid composition: LCGM-2 (AGAS = AlaGlyAlaSer), LCGM-5 (DSGH = AspSerGlyHis), LCGM-10 (KEDV = LysGluAspVal), and LCGM-15 peptide (RAHE = ArgAlaHisGlu). The control groups were treated in parallel with vehicle alone (saline). For drug injection, the fish were anesthetized briefly by placing them in 10°C water. The vehicle controls were tested in parallel (four control groups in total, one for each peptide-treated group).

Behavioral testing was initiated 10 min after drug injection, starting with the novel tank test (NTT) and followed immediately thereafter by the light–dark box (LDB) test. The NTT was adapted from (Maximino et al., 2013) with the behavior video-recorded and the data processed using EthoVision XT14 (Noldus, Netherlands). The distance traveled; speed; the number of visits to the bottom, middle, and top thirds of the aquarium; and the times spent in each of these zones during the initial 5 min in the tank were recorded. A decreased time spent at the surface of the NTT apparatus reflects a reduction in exploratory behavior or increased hiding motivation (Sackerman et al., 2010). The behavior in the LDB test, adapted from (Maximino et al., 2011), was video recorded and processed using EthoVision XT14 (Noldus). The fish were added to the center zone of a three-zone aquarium and allowed to adapt for 1–2 min before removal of the septa separating the center from the flanking zones. The time spent and the number of visits to the light and dark flanking zones and the latency to enter the lit zone were recorded during a 5-min session. Stress of fish is associated with increased time spent in the dark compartment (scototaxis) (Maximino et al., 2011). All behavioral testing was done in the light phase using 500 lux illumination. The experiment was performed by the Institute of Mitoengineering of MSU on a contract basis.

For statistical evaluation, experimental raw data were converted into Z-scores as followed: (1) for each control group mean (μ) and standard deviation (σ) were calculated; (2) Z-score for each fish from the peptide-treated group was found with formula:

*z* $=\frac{x-\mu }{\sigma }$ , where *x* is the observed value in the peptide-treated group, μ and σ are values of the corresponding control group.

### 2.3 Drug treatment and behavioral characterization in rats

We administered LCGM peptides intranasally. Peptides were dissolved in saline at a concentration appropriate for the dosage. For administration, the rat was held in a horizontal position, with its head slightly tilted back. Then, using an automatic laboratory pipette, no more than 10 μL of the peptide solution was carefully introduced into each nostril of the rat. After visually confirming that the entire volume of liquid entered the animal’s nose, the rats were returned to their home cage. The total volume administered did not exceed 20 μL per rat and was calculated based on the animal’s weight. This route of administration has several advantages for short peptides, such as rapid systemic drug absorption and the potential to bypass the blood–brain barrier more effectively and access the central nervous system (Pires et al., 2009; Lochhead and Thorne, 2012). For patients, this method is relatively noninvasive and limits the side effects associated with peripheral administration of substances (Meredith et al., 2015).

Sprague Dawley rats received LCGM-10 or LCGM-15 (1 and 10 mg/kg intranasally in saline) 30 min before testing. The open field (OF) test was used to assess locomotion in a 5-min trial. The round gray polyvinyl chloride OF arena was 97 cm in diameter with 42 cm wall height, and illuminated with bright light at 500 lx (RPC Open Science Ltd). A rat was gently placed in the arena’s center, and the behavior was recorded with a video camera for subsequent analysis by the EthoVision XT videotracking system (Noldus, Netherlands). Measured behavioral parameters were the total distance (cm), the average speed (cm/s), the time spent in the center (s), the number of entries to the center, and the distance traveled in the center (cm). The number of rears and defecation acts were manually counted by the experimenter.

The elevated plus maze (EPM) test was used to assess anxiolytic-like drug activity in a 5-min trial. The maze consisted of two closed and two open arms opposite each other, 30 cm long. The closed arms side height was 15 cm. The entire setup was elevated 70 cm above the floor. The open arms were brightly illuminated at 400 lx, while the closed arms had 30–40 lx (RPC Open Science Ltd). A rat was placed in the maze’s center facing an open arm. The behavior was recorded for analysis with EthoVision XT videotracking system (Noldus, Netherlands), and included the total distance (cm), and the average speed (cm/s). The distance traveled (cm), the time spent (s), and the number of entries were separately calculated for the central sector, open and closed arms.

The interval between sequential behavioral tests was 2–3 days with the sequence OF and EPM. All the testing and data analyses were performed by research personnel blind to treatment. The experiment was performed by the Institute of Mitoengineering of MSU on a contract basis.

### 2.4 Drug treatment and spontaneous locomotor activity assessment in home cage conditions in rats

Spontaneous locomotor activity (SLA) was measured using the Activiscop setup (NewBehavior Inc., Switzerland) (Madani et al., 2003). The animals were kept in home cages with free access to food and water. Motor activity was recorded using an infrared sensor located above each cage. The experiment lasted 2 days: On the first day, baseline activity was recorded, and then Wistar rats received LCGM-10 (5 mg/kg in saline, intranasal) and caffeine (30 mg/kg in saline, intraperitoneal) and were recorded for another 24 h. The days were divided into day 1 (12: 00–18: 59), night (19: 00–06: 59), and day 2 (07: 00–11: 59). The results are presented as the number of behavioral acts per minute (arbitrary units [a.u.]) for each animal. The experiment was performed by the Serbsky National Medical Research Center for Psychiatry and Narcology on a contract basis.

### 2.5 Drug treatment and delay discounting test in rats

The delay discounting test was performed as described previously (Pavlova et al., 2020). Briefly, Wistar rats were first trained to press the two pedals to obtain food: standard food granules weighing 45 mg from BioServ (United States). Throughout the training, rats were maintained at 85% of their free feeding body weight to create food motivation. Training continued until the rats displayed an equal probability of pressing the two pedals. The animals were then presented with the opportunity to select either the low-value immediate reinforcement or the valuable but delayed food reinforcement. During the experiments, the rats performed 25 trials to reach a stability criterion—preference for one pedal or the other—for 10 days. In each experiment the number of presses on the pedal yielding the low-value immediate reinforcement (k1) was determined, along with the number of presses on the pedal leading to delivery of the more valuable but delayed reinforcement (k2). The percent choice of the large/delayed lever (impulsivity coefficient) was calculated as % choice = k2/(k1 + k2). The rats were assigned to the high impulsivity (HI) group if they chose a low-value reinforcement in at least 60% of trials, and to the self-controlling (low-impulsive) group if they were able to choose a more valuable reinforcement in more than 60% of trials. At this stage, 30 HI animals were selected and three groups of 10 rats each were formed. The HI rats first received a single dose of LCGM-10 (1 and 10 mg/kg in saline, intranasal) 30 min before testing. Four days later, the rats received chronic (7-day) administration of LCGM-10 at the doses indicated above, with impulsivity tested the day after the last administration (Table 1). The experiment was performed by the Institute of Higher Nervous Activity and Neurophysiology on a contract basis.

**Table 1:** Schedule of the delay discounting experiments.

Treatment	–	Acute saline	–	Acute LCGM-10/saline	–	Chronic LCGM-10/saline	–
Time interval	1 month	30 min	1 day	30 min	4 days	7 days	–
Delay discounting	Training	Test	–	Test	–	–	Test

### 2.6 Calcium flux imaging

mGluR5 activation leads to Ca^2+^ influx that could be blocked by adding a specific antagonists or NAMs (Kettunen et al., 2002; Beggiato et al., 2018). Elevation of intracellular Ca^2+^ levels via the phospholipase C pathway is typical for all Gq-coupled GPCRs (Liu et al., 2008) and can be detected using a penetrating Ca^2+^ indicator.

The CHO cell line stably expressing human mGluR5 was generated using T-Rex System (Thermo Fisher Scientific Inc., United States) according to the manufacturer’s instructions. Briefly, complementary DNA (cDNA) encoding human mGluR5 was subcloned into the pcDNA4/TO inducible expression vector, which was transfected into CHO cells carrying the pcDNA6/TR regulatory vector that expresses the tetracycline repressor. After 2 weeks of selection using 5 μg/mL blasticidin (Thermo Fisher Scientific Inc.) and 250 μg/mL zeocin (Thermo Fisher Scientific Inc.), pools of cells were screened for the expression of mGluR5 in the agonist-induced Ca^2+^ uptake assay. Positive cells were expanded and used. mGluR5 expression was induced by adding up to 1 μg/mL of tetracycline (Thermo Fisher Scientific Inc.) 16 h before testing.

Fluorescent assays were performed using NOVOstar (BMG LABTECH, Germany). CHO-mGluR5 cells were seeded into black-walled, clear-bottomed 96-well plates at a density of 75,000 cells per well (complete media without antibiotics and containing 1 μg/mL of tetracycline to induce receptor expression) and were cultured overnight at 37°C. The cells were then loaded with the cytoplasmic calcium indicator Fluo-4 AM using the Fluo-4 Direct^™^ Calcium Assay Kits (Thermo Fisher Scientific Inc.) and incubated in the dark at 37°C for 60 min, and then at 25°C for 60 min. The buffer alone (control) or the buffer containing different concentrations of LCGM-10 (0.02, 2, 20, and 200 μM), LCGM-15 (0.02, 2, 20, and 200 μM), or 2-methyl-6-(phenylethynyl) pyridine ([MPEP] 0.1, 1, 10, and 100 μM; Sigma-Aldrich, United States) were added to the cells (in six replicate wells). The LCGM doses 2 and 20 μM are physiologically relevant. After incubation at 37°C for 3 min, changes in cell fluorescence (lex = 485 nM, lem = 520 nM) were monitored before and after the addition of the mGluR5 agonist (1 mM GluNa; Sigma-Aldrich). The measurements were performed at pH 7.4 and 37°C.

### 2.7 Luciferase reporter assay

All GPCR signaling pathways eventually induce gene transcription. Sensitive and easy-to-use high-throughput assays that can accurately detect gene expression activity of a GPCR and validate accurate pharmacology while offering flexibility are based on gene promoter and/or transcription factor response to a GPCR. Ultimately, a chemiluminescent signal is produced by the promoter-driven reporter expression that is directly proportional to activation or inhibition of a specific GPCR in the cells (Unal, 2019).

The detailed description of the method was published previously (Kroeze et al., 2015). Briefly, the HEK293 cell line was transfected with three types of plasmids using polyethylenimine (PEI, 408727, Sigma-Aldrich, United States) 24 h prior to agonist application. Plasmid 1 encodes the GRM5–tTA fusion protein. The linker between mGluR5 and tTA is sensitive to TEV protease.^1^ In case of testing the activity of LCGM-10 peptide toward mGluR1 and mGluR4, we applied the plasmids encoding GRM1-tTA^2^ and GRM4-tTA^3^ fusion proteins, respectively. Plasmid 2 encodes β-arrestin2–TEV protease fusion protein.^4^ Plasmid 3 encodes the luciferase tTA reporter.^5^ Transfected cells were treated with agonist/antagonist and incubated overnight (16 h). Antagonists were introduced 10 min prior to agonists. The following substances were used: α-amino-2-chloro-5-hydroxybenzeneacetic acid, (RS)-2-chloro-5-hydroxyphenylglycine ([CHPG], an orthosteric selective mGluR5 receptor agonist; HB0033, HelloBio, United States) at a dose of 1 mM according to the literature (Loane et al., 2009; Chen et al., 2012). A selective mGluR5 NAM 6-methyl-2-(phenylazo)-3-pyridinol (SIB 1757, Sigma-Aldrich, United States) at a dose of 10 μM according to the literature (Liu et al., 2014), L-(+)-2-amino-4-phosphonobutryic acid ([L-AP4], an orthosteric agonist of Group III mGluRs; HB0370, HelloBio, United States) for mGluR4 activation at a dose of 1 μM according to the literature (Mathiesen et al., 2003), (S)-3,5-dihydroxyphenylglycine ([DHPG], an orthosteric agonist of Group I mGluRs; HB0045, HelloBio, United States) for mGluR1 activation at a dose of 10 μM (Fukuda et al., 2009), (−)-PHCCC (a PAM of mGluR4 and a NAM of mGluR1; SML1432, Sigma Aldrich, USA) at a dose of 40 μM (Maj et al., 2003), and LCGM peptides at doses of 20 and 200 μM according to the results obtained from the Ca^2+^ imaging assay. The luciferase test was performed using Promega^™^ Luciferase Assay Systems Kit (PR-E1500, Promega, United States) according to the manufacturer’s protocol.

### 2.8 Computational studies

We used a computer-generated random tetrapeptide library and experimentally derived peptides from *Bos taurus milk* hydrolysates (supplied by Lactocore Inc.), as described previously (Malyshev et al., 2021) as a source of tetrapeptides. For the discovery of peptidic hits, we utilized the proprietary Peptimize algorithm (Lactocore, Inc.), based on the Peptogrid algorithm (Zalevsky et al., 2019; Malyshev et al., 2021). It is used to post-process a docking run, and as its inputs, we used docking results of all tetrapeptides constructed from 19 amino acids (all canonical residues excluding cysteine). This dataset consisted of 130,321 peptides in total for computer-generated peptides. Additionally, we tested a set of peptides from the hydrolysate: 274 peptides and 5,480 poses in total. We performed docking with AutoDock Vina v. 1.1 (Trott and Olson, 2009) at the NAM site of the 5CGC mGluR5 model (Christopher et al., 2015). We centered the box at the center of masses of the atoms of the NAM site present in the model, with a size of 35 Å in all directions. We set the exhaustiveness parameter was set to 64. The rationale of the docking exclusively to NAM site of mGluR5 is conditioned by the evidence that it provides binding of highly selective allosteric antagonists possessing no affinity for other subtypes of metabotropic glutamate receptors (Christopher et al., 2015).

Due to the narrowness of the binding pocket, peptides with large side groups sterically were not able to fit in it and grouped on the edge of the docking site. We excluded these peptides from further calculations to prevent distortion of the Peptimize probability model. We used 0.92 as a threshold to cut off a sufficient number of the best findings with a good score for the generated set of the peptides. This gave us 20 findings in total that we used for subsequent expert analysis. We also filtered the peptides that originated from *B. taurus* milk hydrolysates. The final ranking had a threshold of 0.74, which provided five peptides for expert analysis. This analysis was focused on finding novel, previously undescribed peptides with patent purity.

### 2.9 Statistical analyses

Statistical analyses were performed using GraphPad Prism 9.5 (GraphPad Software, United States). The *in vitro* luciferase reporter assay was performed in three biological replicates and analyzed with two-way analysis of variance with the *post hoc* Bonferroni multiple comparison test. Ca^2+^ imaging studies were analyzed with one-way analysis of variance, followed by pairwise comparisons using false discovery rate (FDR) correction. *In vivo* screening experiments in *D. rerio* were analyzed by two-way analysis of variance; pairwise comparisons were carried out for each treatment group. The FDR method using two-stage linear step-up procedure of Benjamini, Krieger, and Yekutieli was then applied, with a significance threshold of *q* = 0.05. Behavioral studies in rats (the OF, EPM, SLA, and delay discounting tests) were analyzed using a parametric test (one-or two-way analysis of variance with the *post hoc* Holm–Šídák test) or a non-parametric test (Kruskal–Wallis test with the *post hoc* Dunn’s test) after diagnostics for residuals (Spearman’s rank correlation test for heteroscedasticity and normality tests). Differences between groups were considered significant at *p* < 0.05.

## 3 Results

### 3.1 Docking to the NAM site of mGluR5

We used the proprietary Peptimize engine to obtain a ranked list of tetrapeptide hits against the transmembrane NAM site of mGluR5, also referred to as a MPEP site, similarly to what was done previously (Malyshev et al., 2021). This site is highly selective, it ensures binding of mGluR5 noncompetitive antagonists (MPEP, MTEP, SIB-1757, and their analogs) which do not affect other mGluRs, except of a few exclusions. In some instances, it is reported that MPEP and SIB-1893 are able to act as PAMs of mGluR4 (Mathiesen et al., 2003; Dalton et al., 2017). The effect is not observed in the case of more potent and specific MTEP and SIB-1757. From the overall set of possible tetrapeptides without cysteine, we selected the peptides AGAS, DSGH, and RAHE (hereafter LCGM-2, LCGM-5, and LCGM-15, respectively) for further validation. We chose them because they were ranked in the top 20 list and had significantly different compositions and hence physicochemical properties. In manual selection, we considered interactions with residues of the binding site, chemical diversity, and energy contributions to the binding energy that are not part of the scoring function (primarily ligand strain and binding site desolvation). We also added the best-ranked milk hydrolysate peptide LCGM-10 to the short list.

### 3.2 Behavioral screening of LCGM peptides in *Danio rerio*

**Figure 1:**
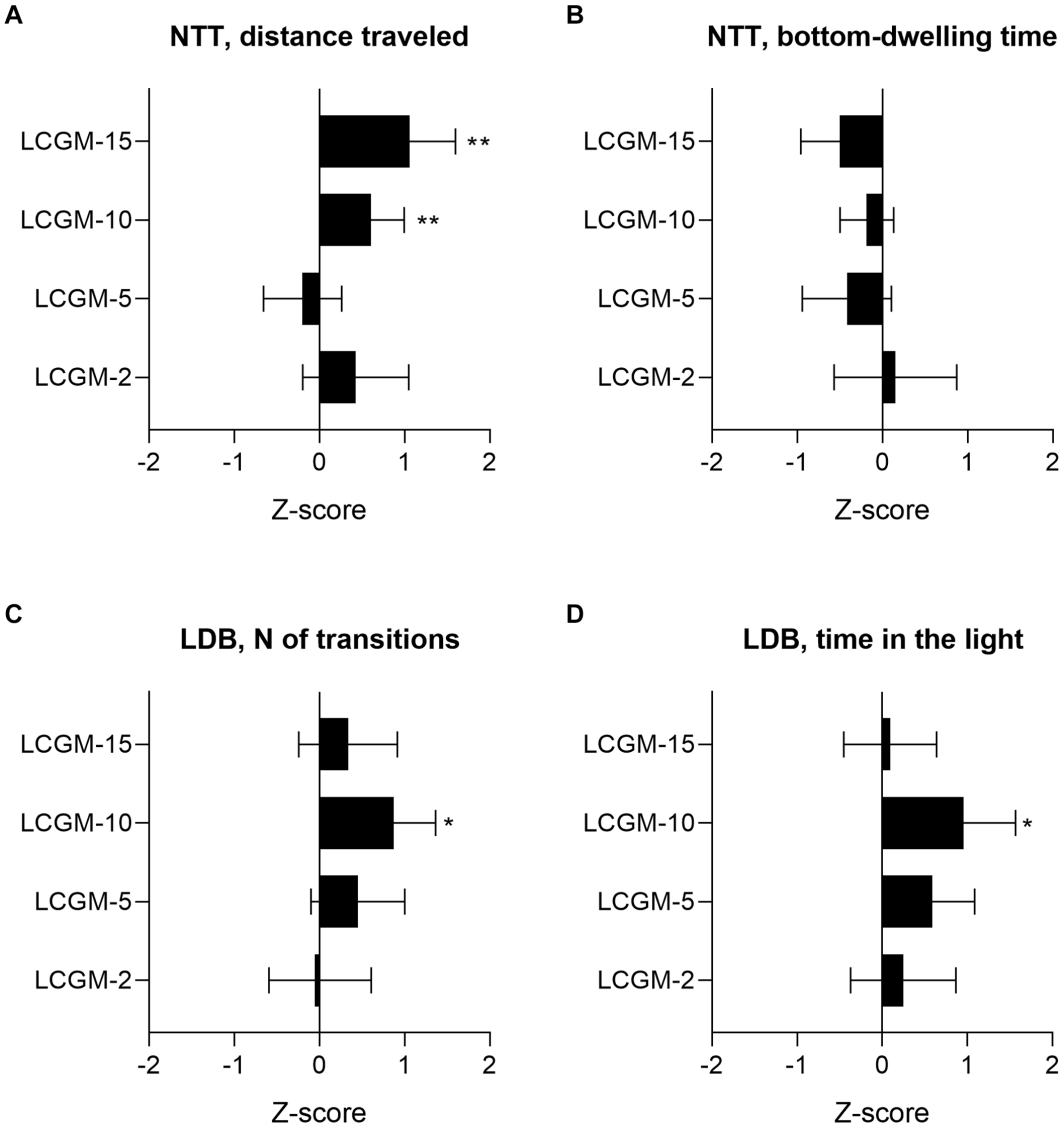
The results of the screening experiment in *D. rerio*. The peptides LCGM-2, LCGM-5, LCGM-10, and LCGM-15 were tested for *in vivo* activity in zebrafish (*N* = 16 in each group) after a single intraperitoneal injection of 1 mg/kg. The bar plots show the effects (z-scores) of the tested peptides in the **(A,B)** NTT and **(C,D)** LDB test. **(A)** Fish locomotor activity was significantly increased after acute LCGM-10 and LCGM-15 administration, but there was no effect from LCGM-2 and LCGM-5 administration. **(B)** The preference of the fish for the tank’s bottom remained unaffected by all of the peptides. **(C)** The number of light-dark transitions and **(E)** the time spent in the light increased significantly in the fish treated with LCGM-10, but not LCGM-2, LCGM-5, and LCGM-15. The results are presented as the mean and 95% confidence interval. *q < 0.05 and **q < 0.01 versus the corresponding control group (not shown); two-way analysis of variance followed by the two-stage linear step-up procedure of Benjamini, Krieger, and Yekutieli, at a q threshold of 0.05. For statistics, see Table 2.

We carried out the two most common tests to evaluate the fish response to the stressful conditions of novelty (NTT) and bright light (LDB). Figure 1 represents the summary data of the effects of the LCGM peptides on the NTT and LDB results relative to each group control, expressed as z-scores. The Supporting Information contains the examples of motor tracks (Supplementary Figures S1, S2), and primary data (Supplementary Figures S3, S4), and Table 2 provides the results of the two-way ANOVA. Statistical analysis revealed a significant effect for Treatment (*F*_1, 124_ = 7.8, *p* = 0.005) and Treatment × Group interaction (*F*_3, 124_ = 4.4, *p* = 0.05) on the distance traveled by the fish, but not the time spent on the bottom in the NTT. LCGM-10 and LCGM-15 had a stimulating effect. Both peptides delivered intraperitoneally at 1 mg/kg significantly increased the distance traveled by the fish (*q* = 0.004 for LCGM-10, and *q* = 0.005 for LCGM15; Figure 1A), with no major changes in the bottom-dwelling time (Figure 1B). In the LDB test, a significant Treatment effect was found for the number of transitions (*F*_1, 124_ = 6.3, *p* = 0.013) and the time spent in the light (*F*_1, 124_ = 7.4, *p* = 0.007) by the fish. We observed an increase in the number of transitions to the light (*q* = 0.018; Figure 1C) as well as the time spent in the light compartment (*q* = 0.022; Figure 1D) after treatment with LCGM-10, suggesting potential anxiolytic-like activity that we have described previously for diazepam (Malyshev et al., 2021). LCGM-2 and LCGM-5 had no effect on the behavior of the fish in either test.

**Table 2:** The results of the statistical analysis of *D. rerio* behavior in the NTT and LDB test.

Two-way analysis of variance	*F* (DFn, DFd)	*p*
**NTT, distance traveled**		
Interaction	*F*_3, 124_ = 4.4	0.005*
Treatment	*F*_1, 124_ = 7.8	0.005*
Group	*F*_3, 124_ = 2.4	0.062
**NTT, bottom-dwelling time**		
Interaction	*F*_3, 124_ = 0.7	0.538
Treatment	*F*_1, 124_ = 2.6	0.103
Group	*F*_3, 124_ = 6.3	0.001*
**LDB test, number of transitions**		
Interaction	*F*_3, 124_ = 1.3	0.260
Treatment	*F*_1, 124_ = 6.3	0.013*
Group	*F*_3, 124_ = 1.3	0.250
**LDB test, time in the light**		
Interaction	*F*_3, 124_ = 1.3	0.251
Treatment	*F*_1, 124_ = 7.4	0.007*
Group	*F*_3, 124_ = 0.5	0.665

We carried out two-way analysis of variance with the independent variables (factors) treatment (control or peptide) and group (LCGM-2, LCGM-5, LCGM-10, or LCGM-15).

*p < 0.05.

### 3.3 Calcium flux imaging of LCGM peptides

**Figure 2:**
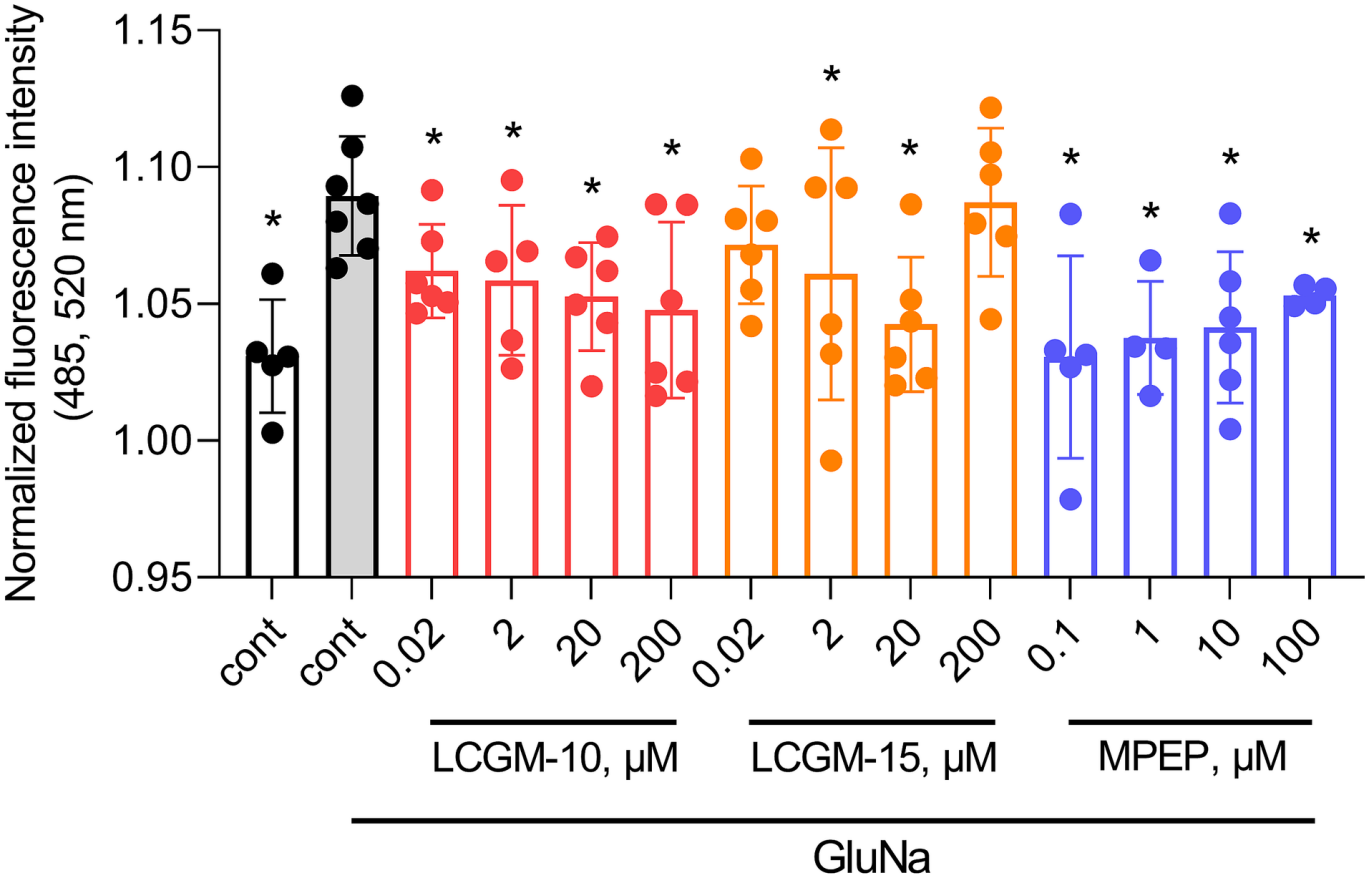
Effects of LCGM-10 and LCGM-15 on intracellular Ca^2+^ levels in CHO-mGluR5 cells at peak activation (maximum fluorescence amplitude). Basal fluorescence [cont, before activation (*N* = 5)] is shown as a white bar. The mGluR5 agonist GluNa (1 mM) enhanced Ca^2+^ influx (activated cont, gray bar, *N* = 7). There was a significant effect on intracellular Ca^2+^ levels with 0.02–200 μM LCGM-10 (*N* = 6 each), 2 and 20 μM LCGM-15 (*N* = 6 each), and 0.1–100 μM of the mGluR5 antagonist MPEP (*N* = 6 each) (*F*_13, 64_ = 2.8, *p* = 0.003; one-way analysis of variance followed by the two-stage linear step-up procedure of Benjamini, Krieger, and Yekutieli, at a q threshold of 0.05). *q < 0.05 versus activated cont. The data are presented as the mean ± standard deviation.

Figure 2 shows the effects of the LCGM peptides and selective non-competitive mGluR5 antagonist MPEP at peak activation in a CHO cell line stably expressing human mGluR5. There was a significatnt Treatment effect with *F*_13, 64_ = 2.8 (*p* = 0.003), and GluNa resulted in an enhanced fluorescence peak intensity compared to non-activated cells (*q* = 0.001). MPEP effectively blocked GluNa-evoked calcium oscillations in cells at all tested doses (0.1 μM with *q* = 0.001, 1 and 10 μM with *q* = 0.004, 100 μM with *q* = 0.03). We found a potent reduction in calcium influx after the application of LCGM-10 at all tested doses (0.02, 2 μM with *q* = 0.04, 20 μM with *q* = 0.02, and 200 μM with *q* = 0.007) and LCGM-15 at doses of 2 μM (*q* = 0.04) and 20 μM (*q* = 0.004), confirming the potential of LCGM peptides as mGluR5 NAMs.

### 3.4 Luciferase reporter assay of the LCGM peptides

**Figure 3:**
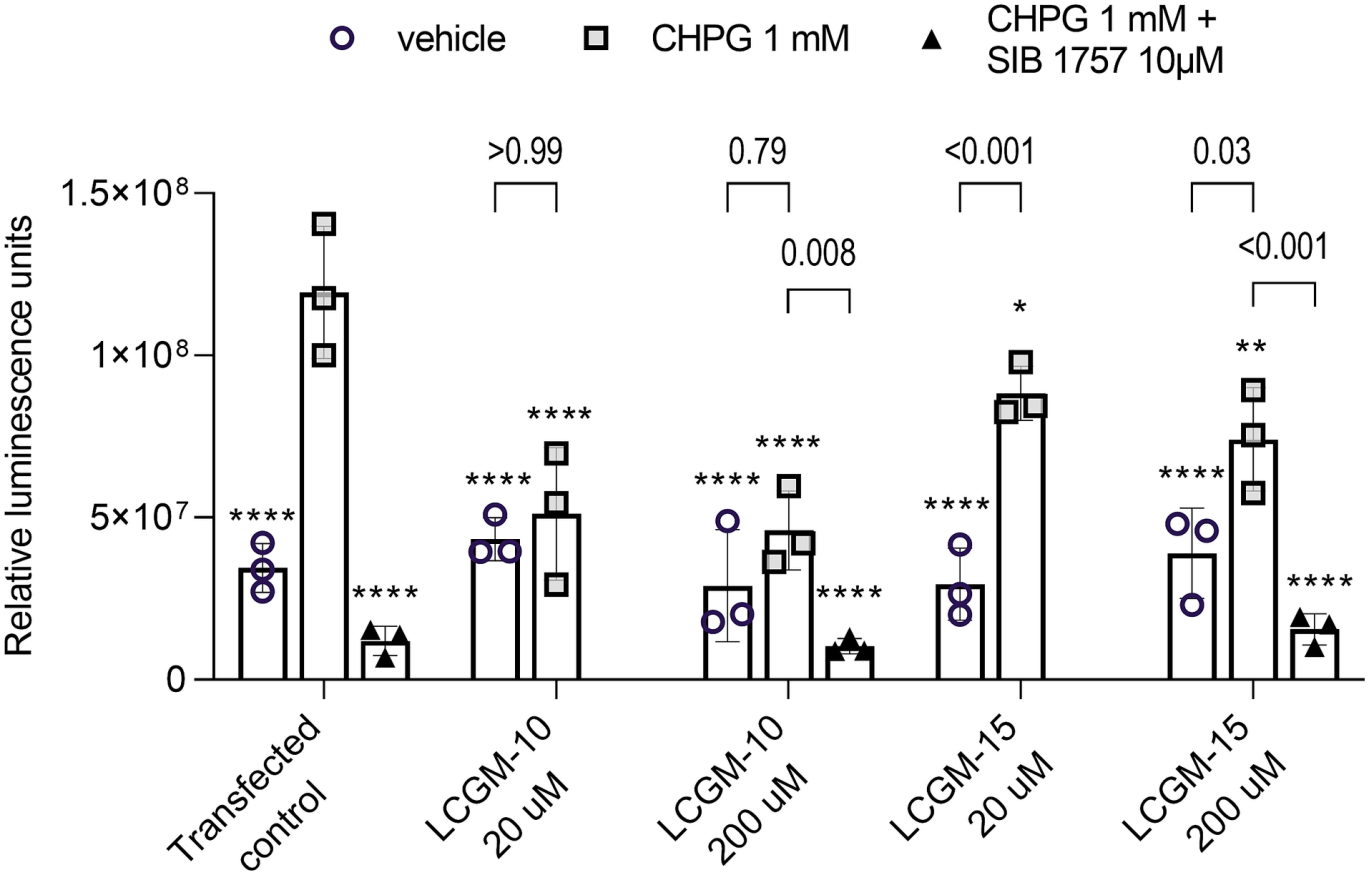
The mGluR5-luciferase reporter assay results. HEK293 cells transfected with genetic reporter systems were treated with CHPG (1 mM) or CHPG (1 mM) + SIB 1757 (10 μM). LCGM-10 and LCGM-15 were co-administered at a dose of 20 and 200 μM. Introduction of the mGluR5 agonist CHPG induced a luciferase signal, corresponding to mGluR5 activation. The mGluR5 antagonist SIB 1757 inhibited mGluR5 activity. Both LCGM-10 and LCGM-15 reduced the level of CHPG-induced mGluR5 activation (Treatment [*F*_1, 20_ = 61.7, *p* < 0.001]; Group [*F*_4, 20_ = 6.41, *p* = 0.002]; Interaction [*F*_4, 20_ = 7.34, *p* < 0.001]; two-way analysis of variance with the *post hoc* Bonferroni multiple comparison test). The data are presented as the mean ± standard deviation for three biological replicates (*N* = 3 each group). *p < 0.05, **p < 0.01, and ****p < 0.0001 versus the transfected control + CHPG 1 mM group. The *p*-values represent the influence of the mGluR5 agonist/antagonist treatment within the groups.

We detected the luciferase signal after 16 h of incubation with LCGM-10 and LCGM-15 (20 and 200 μM) alone, or in combination with a mGluR5 agonist CHPG (1 mM) or CHPG + a mGluR5 antagonist SIB 1757 (10 μM) (Figure 3). Two-way ANOVA revealed a significant effect for Treatment (*F*_1, 20_ = 61.7, *p* < 0.001), Group (*F*_4, 20_ = 6.41, *p* = 0.002), and Treatment × Group interaction (*F*_4, 20_ = 7.34, *p* < 0.001). Transfected control cells luminescence was enhanced in the presence of CHPG (*p* < 0.0001) and depleted when SIB1757 was added (*p* < 0.0001), supporting mGluR5-dependent signal transduction.

LCGM-10 and LCGM-15 reduced the level of CHPG-induced mGluR5 activation compared with the activated control (LCGM-10 at 20 and 200 μM with *p* < 0.0001, and LCGM-15 with *p* = 0.05 and 0.0012 at 20 and 200 μM respectively). In the presence of SIB 1757, the signal was indistinguishably low in all peptide-treated groups and transfected contol. Luminescence signal depression was more evident with LCGM-10 treatments, with no difference from non-activated peptide-treated controls (*p* > 0.79 for 20 and 200 μM), when LCGM-15 application still caused a significant signal enhancement in the presence of CHPG (with *p* < 0.001 at 20 μM, and *p* = 0.03 at 200 μM). Even high doses of LCGM-10 and LCGM-15 did not influence mGluR5 activity in the absence of CHPG.

To exclude off-target activity toward mGluR4, we tested the ability of LCGM-10 peptide to affect the basal activity of mGluR4 and to influence the receptor’s response to a specific agonist L-AP4. Additionally, we checked the activity of LCGM-10 toward mGluR1 which belongs to the same Group I of metabotropic glutamate receptors, utilizing the identical intracellular signaling pathway. LCGM-10 performed no action on both mGluR4 and mGluR1 (Supplementary Figure S5) suggesting the selectivity for mGluR5 over the most relevant mGluR subtypes.

### 3.5 Behavioral analyses of LCGM-10

**Figure 4:**
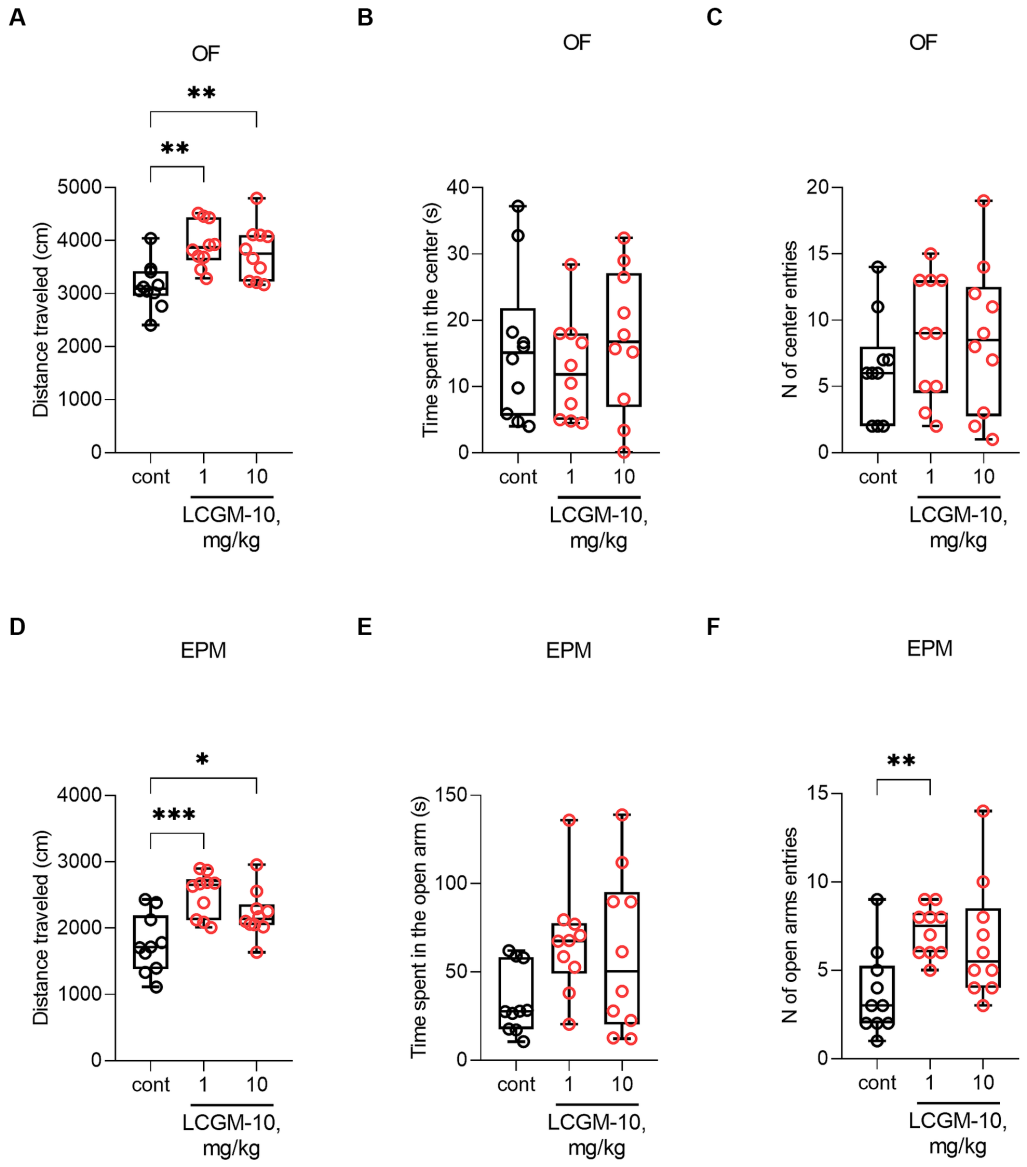
The effects of LCGM-10 administration on the behavior of Sprague Dawley rats in the OF and EPM tests. LCGM-10 was administered intranasally at 0.1 and 1 mg/kg 30 min prior to the test. **(A–C)** In the OF test, LCGM-10 at both doses significantly increased the distance traveled ([*F*_2, 27_ = 7.8, *p* = 0.002]; analysis of variance, with the *post hoc* Holm–Šídák test) without affecting the time spent in the center ([*F*_2, 27_ = 0.5, *p* = 0.61]; analysis of variance) and the number of center entries ([*F*_2, 27_ = 0.8, *p* = 0.46]; analysis of variance). **(D–F)** In the EPM, LCGM-10 significantly increased locomotion at both doses ([*F*_2, 27_ = 9.9, *p* < 0.001]; analysis of variance, with the *post hoc* Holm–Šídák test). LCGM-10 increased the number of transitions to the open arm significantly at 0.1 mg/kg and numerically at 1 mg/kg ([*H*_2, 27_ = 9.9, *p* = 0.007]; Kruskal-Wallis test, with the *post hoc* Dunn’s test), without an overall effect on the time spent in the open arms ([*F*_2, 27_ = 2.8, *p* = 0.07]; analysis of variance). *N* = 10 in each group. *p < 0.05, **p < 0.01, and ***p < 0.0001 versus the control group. The results are presented with box and whisker plot.

Analysis of the behavioral data revealed a significant Treatment effect on distance traveled in the OF (*F*_2, 27_ = 7.8, *p* = 0.002) and the EPM (*F*_2, 27_ = 9.9, *p* < 0.001) tests. Track visualizations are presented in the Supplementary (Supplementary Figure S6 for the OF, Supplementary Figure S7 for the EPM). Rats treated intranasally with 1 and 10 mg/kg LCGM-10 showed increased locomotor activity in both OF (*p* = 0.002 and 0.006 respectively) and EPM tests (*p* < 0.001 and *p* = 0.01 respectively) (Figure 4). In the OF, the difference was found in the distance traveled near the walls (*F*_2, 27_ = 0.79, *p* = 0.002; Supplementary Table S1), but not in the center of the arena (*F*_2, 27_ = 0.07, *p* = 0.93; Supplementary Table S1) after the peptide treatment. LCGM-10 at both doses did not affect thigmotaxis in the OF: The time spent in the center of the arena (*F*_2, 27_ = 0.5, *p* = 0.61) and the number of center entries (*F*_2, 27_ = 0.8, *p* = 0.46) did not differ from the control rats. In the EPM, alongside the increased distance traveled, we observed an increase in the number of open arm entries (*H*_2, 27_ = 9.9, *p* = 0.007; at 0.1 mg/kg with *p* = 0.004). The lack of an effect of LCGM-10 on the time spent in the open arms (*F*_2, 27_ = 2.8, *p* = 0.07) in the EPM and on the time spent and distance traveled in the center of the OF test, suggests that the peptide has a locomotor rather than anxiolytic-like effect.

### 3.6 The effect of LCGM-10 on SLA

**Figure 5:**
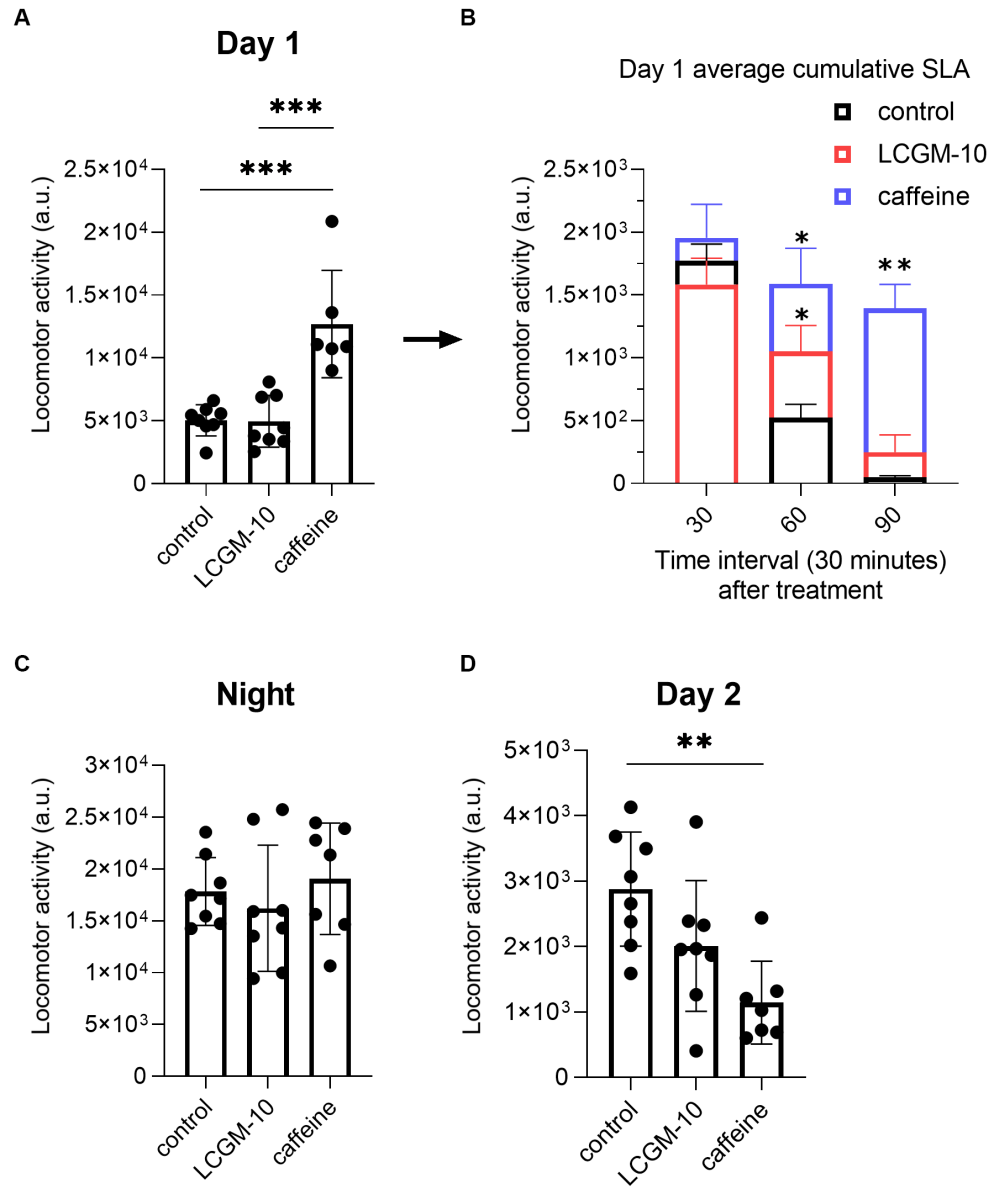
Home cage cumulative SLA in male Wistar rats. Twenty-four-hour recording (day 1 [12:00–18:59], night [19:00–06:59], and day 2 [07:00–11:59]) after treatment with LCGM-10 (5 mg/kg, intranasal) and caffeine (30 mg/kg, intraperitoneal). **(A)** There was significant induction of SLA on day 1 in caffeine-treated group compared with the vehicle-treated control group ([*F*_2, 20_ = 18.7, *p* < 0.001]; one-way analysis of variance with the *post hoc* Holm–Šídák multiple comparison test). **(B)** Day 1 average cumulative SLA after LCGM-10 and caffeine treatment over 30-min intervals. In the short term, there was an increase in SLA in the LCGM-10 group up to 60 min, while caffeine-induced hyperlocomotion was evident up to 90 min (Treatment [*F*_2, 20_ = 12.4, *p* < 0.001]; Time [*F*_2.056, 37.00_ = 92.2, *p* < 0.001], Interaction [*F*_6, 54_ = 6.3, *p* < 0.001]; two-way repeated measures analysis of variance with the *post hoc* Holm-Šídák multiple comparisons test). **(C)** There were no effects of treatment on the nighttime activity in the rats ([*F*_2, 20_ = 0.6, *p* = 0.56]; one-way analysis of variance). **(D)** There was a significant reduction in SLA in the caffeine-treated group compared with the vehicle-treated control group on day 2 ([*F*_2, 20_ = 7.6, *p* = 0.003]; one-way analysis of variance with the *post hoc* Holm–Šídák multiple comparison test). *N* = 7–8 in each group. **p < 0.01 and ***p < 0.001 versus the control group. The bar plots show the mean ± standard deviation.

We measured home cage activity 24 h before (baseline) and after (treatment) drug administration. We divided the 24-h period into day 1 (12:00–18:59), night (19:00–06:59), and day 2 (07:00–11:59). Baseline activity in rats was similar between the groups (not shown). There was enhanced SLA in rats on day 1 after injection of 30 mg/kg caffeine (*F*_2, 20_ = 18.7, *p* < 0.001; *p* < 0.001 vs. control group) followed by hypokinesia on day 2 (*F*_2, 20_ = 7.6, *p* = 0.003; *p* = 0.002 vs. control group) (Figures 5A,D). There were no differences between the groups during the night (*F*_2, 20_ = 0.6, *p* = 0.56) (Figure 5C). LCGM-10 (5 mg/kg) did not cause an overall shift in diurnal activity in rats. We performed a thorough analysis of day 1 to determine whether the peptide caused a short-term locomotor effect. Figure 5B shows SLA during the first 1.5 h after treatment, averaged for every 30 min with significant Treatment (*F*_2, 20_ = 12.4, *p* < 0.001), Time effects (*F*_2.056, 37.00_ = 92.2, *p* < 0.001), and their Interaction (*F*_6, 54_ = 6.3, *p* < 0.001). After intranasal administration of LCGM-10, the rats maintained greater locomotor activity compared with the vehicle-treated control group for a relatively short period of time: the total 30-min locomotor activity was higher in the LCGM-10 group at 60 min (*p* = 0.04) but not at 90 min (*p* = 0.20).

### 3.7 The effects of LCGM-10 in the delay discounting test

**Figure 6:**
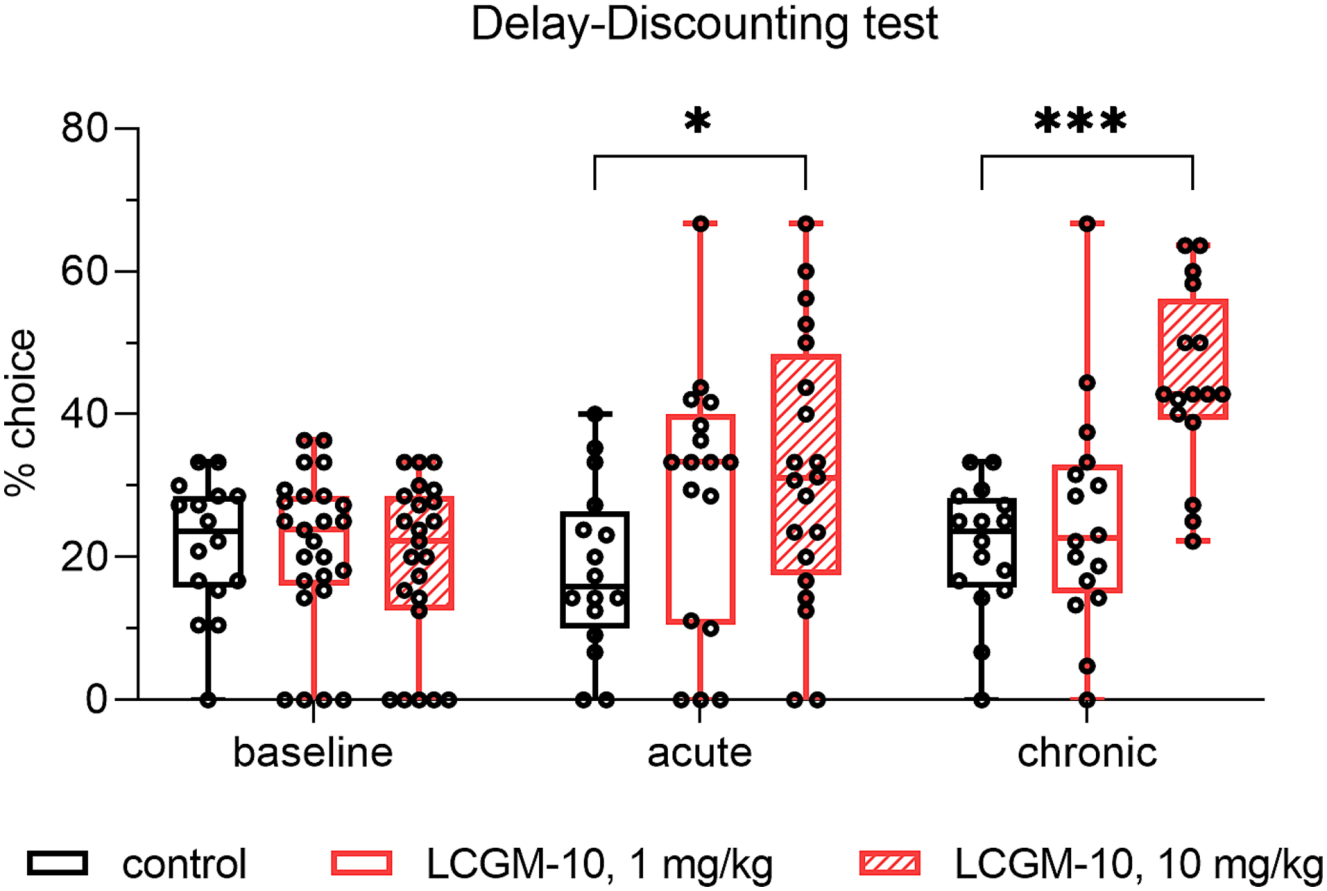
Percent choice of the large/delayed lever in the delay discounting test in male Wistar rats. HI rats received a single dose (acute administration) or chronic doses (7-day administration) of LCGM-10 at 1 and 10 mg/kg. Acute and chronic administration of 10 mg/kg LCGM-10 was sufficient to promote choosing the large/delayed lever (Group [*F*_2, 61_ = 8.2, *p* < 0.001], Treatment [*F*_1.681, 79.84_ = 7.8, *p* = 0.002], Interaction [*F*_4, 95_ = 5.3, *p* < 0.001]; two-way repeated measured analysis of variance with the *post hoc* Holm–Šídák multiple comparison test). Control groups *N* = 16 each, peptide-treated groups (acute) *N* = 17–20, peptide-treated groups (chronic) *N* = 23–25. The results are presented with a box and whisker plot.

Investigation of acute and chronic LCGM-10 administration on trait impulsivity of highly impulsive rats revealed reduced delay aversion in the delay discounting test with Group (*F*_2, 61_ = 8.2, *p* < 0.001), Treatment (*F*_1.681, 79.84_ = 7.8, *p* = 0.002), and Interaction effects (*F*_4, 95_ = 5.3, *p* < 0.001). We trained male Wistar rats to press levers to receive either an immediate small food reward or a delayed larger reward. We considered rats with >60% preference for an immediate reward to be impulsive and we divided them into three groups treated with saline (control) or LCGM-10 at a dose of 1 or 10 mg/kg. Baseline selection of a large/delayed reward after intranasal saline administration did not differ between the groups (Figure 6). We found that acute and chronic intranasal administration of LCGM-10 at a higher dose increased the proportion of large/delayed lever presses (*p* = 0.02 and *p* < 0.001 for acute and chronic treatment respectively), which suggests decreased reward choice impulsivity after treatment with this peptide.

## 4 Discussion

In the current study we describe the discovery of LCGM-10 peptide, a potential mGluR5 NAM drug. LCGM-10 was among the top-ranked peptides according to the docking studies. *In vivo* behavioral screening in *D. rerio* revealed stimulating activity of LCGM-10, which were further supported in the behavioral test in rats. In depth analysis of locomotor effects of LCGM-10 revealed moderate enhancement of SLA in rats evident up to a 60 min after administration. We also found that LCGM-10 acute and chronic administration potently decreases impulsive choice in rats in the delay discounting test. LCGM-10 acted as NAMs in functional assays, reducing calcium ion flux in CHO-mGluR5 cells activated with GluNa and suppressing luciferase signal when co-administered with mGluR5 agonist CHPG. These results suggest a potential implications of mGluR5 NAM LCGM-10 for the treatment of movement disorders, ADHD, OCD, and pathological conditions associated with impulsive behavior (drug addiction and gambling).

We evaluated the *in vivo* activity of the top-ranked peptides from the docking investigation in zebrafish. *D. rerio* is a valuable screening tool for pharmacological studies as they respond to psychoactive compounds of various classes, such as the GABAA PAM diazepam; the 5-HT_1A_ receptor agonist buspirone; the serotonin–norepinephrine reuptake inhibitor desipramine; and the selective serotonin reuptake inhibitors fluoxetine, caffeine, and ethanol (Maximino et al., 2013, 2014; Cueto-Escobedo et al., 2022). Our previous screening study of peptides with anxiolytic-like and antidepressant-like properties showed a specific activity profile in zebrafish, similar to what has been observed after diazepam treatment (Malyshev et al., 2021). Herein, we found the stimulatory effects for two peptides, LCGM-10 and LCGM-15. A previous report indicated a similar effect of low-dose caffeine (10 mg/kg) and bupropion (30 mg/kg) on locomotion; these drugs have known motor-stimulating effects (Maximino et al., 2014). Based on these results, we chose LCGM-10 and LCGM-15 for further investigation of biological activity and target validation.

Target validation experiments suggested that LCGM-10 has greater efficacy than LCGM-15. LCGM-10 behaved as an mGluR5 NAM *in vitro* as evidenced by its ability to diminish the stimulating effect of GluNa expressed as the maximum fluorescence amplitude in the calcium imaging assay. We observed a significant reduction of calcium influx starting from 0.02 μM up to 200 μM LCGM-10. A similar magnitude of suppression was registered after mGluR5 antagonist MPEP treatment at a dose range of 0.1–10 μM. We next evaluated the modulating effect of LCGM-10 on mGluR5 activity by performing a luciferase reporter gene assay. This finding indicates that both peptides could act as mGluR5 NAMs. LCGM-10 suppressed mGluR5 activity more potently: the luminescence signal did not differ from the peptide-treated non-activated group at either tested LCGM-10 dose in the presence of CHPG, while the signal was higher after treatment with both doses of LCGM-15 compared to non-activated peptide-treated cells. Additionally, LCGM-10 specificity over the other subtypes of metabotropic glutamate receptors was supported by the absence of the activity toward the most structurally close mGluR1 and mGluR4.

Therefore, we evaluated the potential of LCGM-10 as a therapeutic in male rats submitted to the OF and EPM. Notably, the stimulatory effect of the LCGM peptides in zebrafish was also evident in rodent OF and EPM behavioral tests, which suggests evolutionary conservation of the behaviorally relevant target of LCGM-10 across most vertebrates. The importance of mGluR5 for modulating motor behavior has been reported in pharmacological (Mcgeehan et al., 2004; Guimaraes et al., 2015) as well as knockout (Gray et al., 2009; Jew et al., 2013; Ribeiro et al., 2014) studies in rodents, with solid agreement that mGluR5 blockade produces hyperkinesia. The effect of mGluR5 blockade probably depends on the brain region and might involve the cross-interaction of different neural substrates (Jew et al., 2013; Guimaraes et al., 2015). Interestingly, the stimulatory effect on locomotion has been described for the mGluR5 antagonist MPEP, when it was applied directly to olfactory bulbs, the dorsolateral striatum, and the dorsal hippocampus, but not primary motor area and ventral striatum, suggesting an intricate interplay between neural circuits involved in mGluR5-mediated motor behavior regulation (Guimaraes et al., 2015).

To begin exploring the potential of LCGM-10 as a CNS stimulant, we studied SLA in rats. A previous report indicated that 25 mg/kg caffeine increased SLA up to 3 h in mice (Kobayashi et al., 2020), but no studies had investigated long-term effects of single caffeine administration. We found that the locomotor stimulatory effect of LCGM-10 returns to control levels relatively shortly, while caffeine-induced activation is followed by a locomotor depression. Considering the profile of action of the novel peptide, we believe that LCGM-10 might substantially aid in the treatment of hypo-and bradykinesias of various etiologies.

Previous studies have reported that mGluR5 PAMs but not NAMs decrease pharmacologically evoked state impulsivity and preexisting trait impulsivity efficaciously (Isherwood et al., 2015). Two other reports found no effects of mGluR5 antagonists on impulsivity, whereas an mGluR1 antagonist potently mediated the impulsive choice of rats (Sukhotina et al., 2008; Yates et al., 2017). Differences in the methodologies might explain the discrepant results regarding the effects of mGluR modulators in the current work compared with other reports. Same contradictions were previously found for stimulant drugs d-amphetamine and methylphenidate in the delay discounting test, which mostly depend on procedure modifications (Dalley and Roiser, 2012). At the same time, stimulants have been approved by the Food and Drug Administration (FDA) to reduce the core symptoms of ADHD, namely inattention, hyperactivity, and impulsivity.

Delay discounting tasks are also used to assess impulsive behavior in human studies, giving an insight into causal mechanism of addictions such as substance abuse and gambling (Reynolds, 2006; de Wit, 2009; Hodgins and Holub, 2015), and ADHD (Barkley et al., 2001; Scheres et al., 2008; Shiels et al., 2009). Impulsivity in the human and rodent delay discounting task derives form the similar neural substrates: lower D2/3 receptor availability in ventral striatum of rats and pathological gamblers, individuals with methamphetamine dependence, and alcohol use disorder (Voon and Dalley, 2015). Lesions of the medial orbitofrontal cortex (OFC) increased delay discounting in rats (Mar et al., 2011), and similarly, stroke-induced lesions of the medial OFC increased delay discounting in humans (Sellitto et al., 2010). Translational potential of rodent delay discounting test is also supported by the studies of currently marketed medications. For instance, methylphenidate, a stimulant approved by the FDA and the first-choice drug treatment for ADHD, reduced discounting of delayed rewards in both human (Shiels et al., 2009) and animal models (Wooters and Bardo, 2011). The results obtained for LCGM-10, and the published data suggests promising translational potential of the peptide for the treatment of conditions associated with maladaptive impulsivity. At the same time, to obtain more solid evidence of therapeutic potential for certain disorders, future studies in more specific animal models are required.

Although the main systems regulating impulsive behavior are the 5-HT and DA circuitry (Winstanley et al., 2006; Pattij and Vanderschuren, 2008; Dalley and Roiser, 2012), glutamate also plays an important role in this process. Metabotropic glutamate receptors modulate impulsivity in animal models (Pattij and Vanderschuren, 2008). Moreover, mGluR5 functionally interacts with N-methyl-D-aspartate (NMDA) receptors, and their activity can modulate synaptic plasticity (Simonyi et al., 2005) and thus affect impulsive control. In addition to the effects on glutamate and GABA function, NMDA receptor antagonists and mGluR modulators may interact at the level of the mesolimbic and mesocortical dopamine systems (Isherwood et al., 2015). Recent studies have also found increased impulsivity, risky decision-making, and reward-system dysfunction in patients with OCD, features that are usually linked to the development of substance and behavioral addictions (Balogh et al., 2013; Benatti et al., 2014; Grant and Chamberlain, 2014; Grassi et al., 2015).

To sum up, the development of new medications for disorders with an excessive impulsivity as a major component is of high interest for both academy and industry. Addictions, including substance use disorder and gambling, faces the unmet need for novel medicines (Rash et al., 2016). In case of ADHD there is also a need for new medications with novel mechanisms of action since currently approved drugs show a non-response or lack efficacy in a significant number of patients (Mechler et al., 2022). Routinely used stimulant methylphenidate is efficient in up to 70% of cases (Bodey, 2011), and the effect sizes of the most frequently prescribed non-stimulants atomoxetine, clonidine and guanfacine are generally in the medium range not exceeding 60% and smaller than those of stimulants (Cortese et al., 2018). Concluding, the discussed disorders remain a promising niche that demands the development of a novel drug candidates.

## 5 Conclusion

In summary, LCGM-10 acts as an mGluR5 NAM and produces effects like those of stimulants. The enhanced locomotor activity produced by LCGM-10 suggests the peptide could be used to treat hypo-and bradykinesias, which are observed in patients with Parkinson’s disease and other diseases affecting the basal ganglia, and are also adverse effects of antipsychotic and antidepressant medications. The reduction in trait impulsivity by LCGM-10 suggests the peptide could be used to treat ADHD, OCD, and some maladaptive behaviors associated with increased impulsivity. Additional characterization of LCGM-10 will be necessary to ascertain its target as well as its cellular mechanism of action and therapeutic potential.

Alongside further mechanistic studies, the future direction of our work will include more deeply understanding the structure–activity relationships of LCGM-10. We will also conduct pharmacokinetics and absorption, distribution, metabolism, and excretion studies to determine its degradation profile, and optimize it through chemical modifications for metabolic and related drug-like properties.

## Data availability statement

The raw data supporting the conclusions of this article will be made available by the authors, without undue reservation.

## Ethics statement

Ethical approval was not required for the studies on humans in accordance with the local legislation and institutional requirements because only commercially available established cell lines were used. The animal study was approved by Institute of Mitoengineering of MSU and Institute of Higher Nervous Activity and Neurophysiology local Bioethics Commissions. The study was conducted in accordance with the local legislation and institutional requirements.

## Author contributions

AM: Conceptualization, Data curation, Project administration, Supervision, Writing – review & editing. VP: Conceptualization, Data curation, Investigation, Methodology, Supervision, Writing – original draft. NM: Conceptualization, Data curation, Investigation, Methodology, Supervision, Writing – original draft. IS: Data curation, Formal analysis, Visualization, Writing – original draft, Writing – review & editing. VG: Formal analysis, Investigation, Methodology, Writing – original draft. AZ: Data curation, Investigation, Software, Writing – original draft. ID: Conceptualization, Project administration, Resources, Supervision, Writing – review & editing. GB: Funding acquisition, Project administration, Resources, Writing – review & editing. TS: Conceptualization, Writing – review & editing.

## Footnotes

^1^ https://www.addgene.org/66390/

^2^ https://www.addgene.org/66387/

^3^ https://www.addgene.org/66389/

^4^ https://www.addgene.org/107245/

^5^ https://www.addgene.org/64127/
