# Supplementary material for: The novel peptide LCGM-10 attenuates metabotropic glutamate receptor 5 activity and demonstrates behavioral effects in animal models
Source: Front Behav Neurosci. 2024 Feb 7;18:1333258. doi: 10.3389/fnbeh.2024.1333258 (PMC10879279; doi:10.3389/fnbeh.2024.1333258)
Supplement: Supplementary file 1 [file Data_Sheet_1.docx]

Supplementary Material


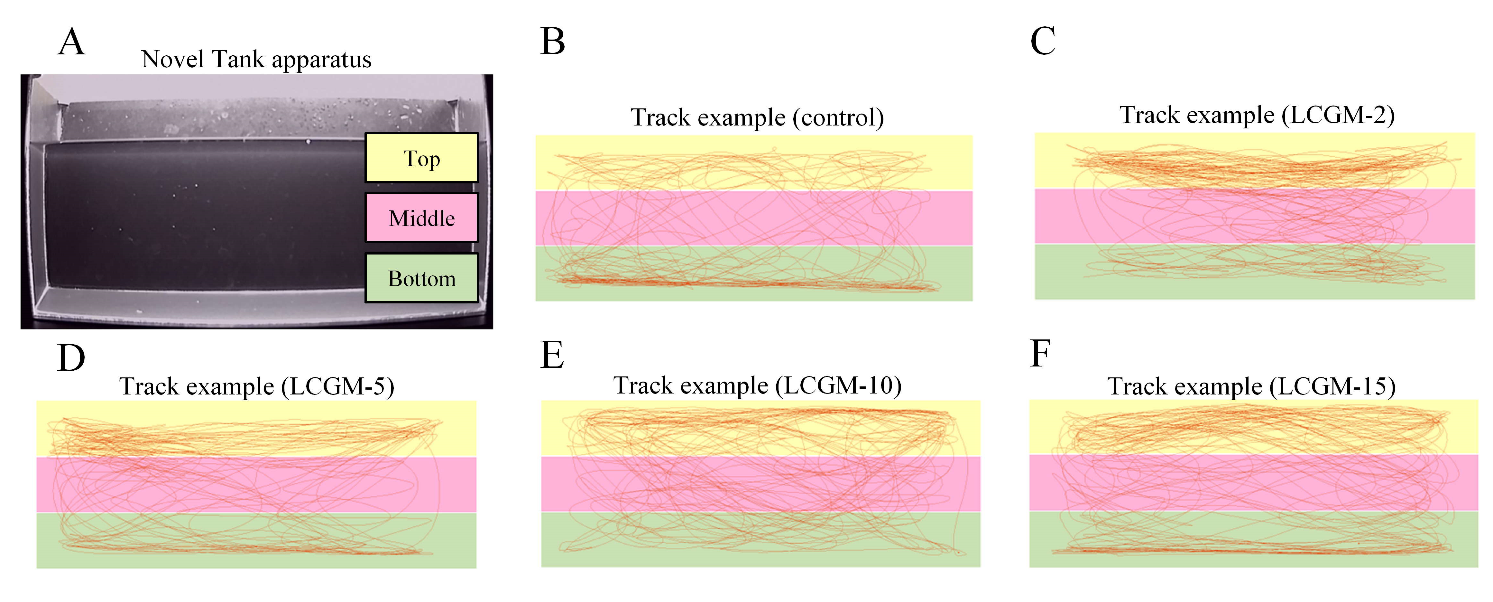


**Figure S1**. **Novel tank test.** General view of the arena (**A**). **Animal track visualization.** Examples of tracks: Control (B); LCGM-2 (C); LCGM-5, (D); LCGM-10 (E); LCGM-15 (F).


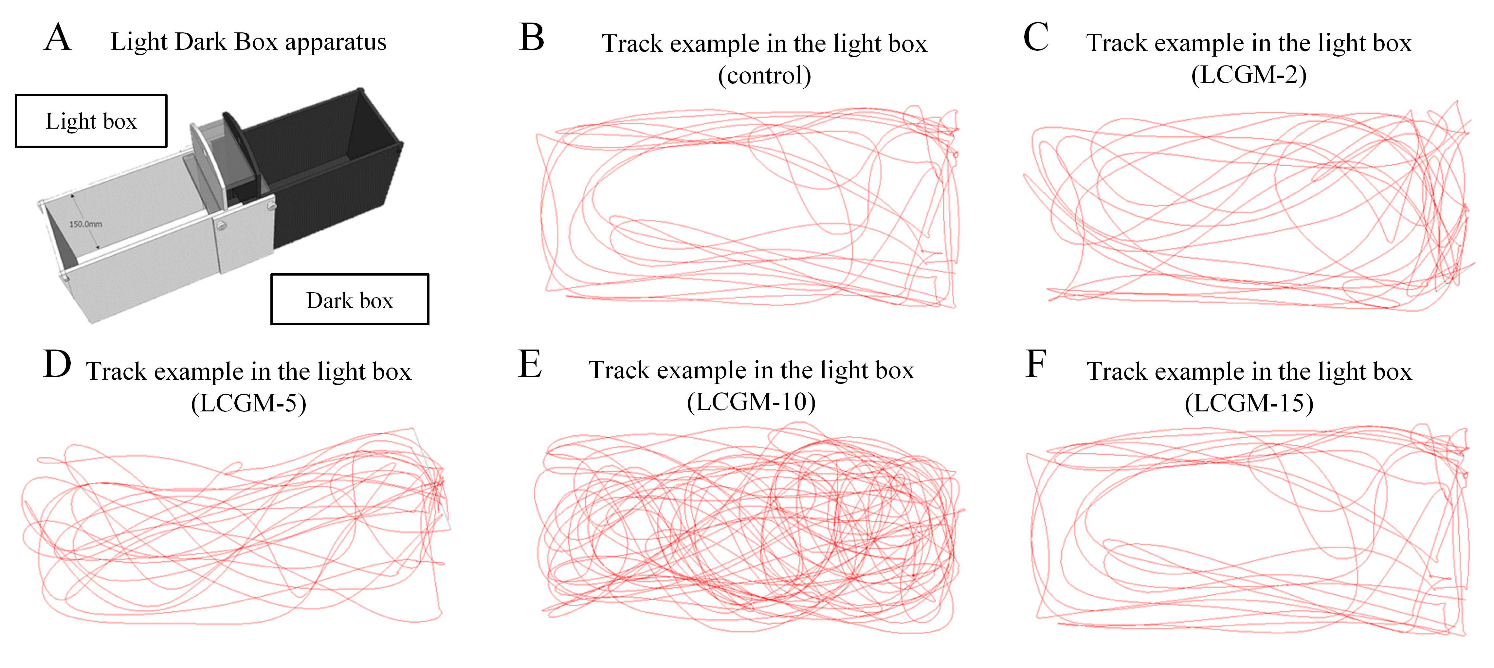


**Figure S2**. **Light-dark box test.** General view of the arena (**A**). **Animal track visualization in the light box.** Examples of tracks in light box: Control (B); LCGM-2 (C); LCGM-5, (D); LCGM-10 (E); LCGM-15 (F).


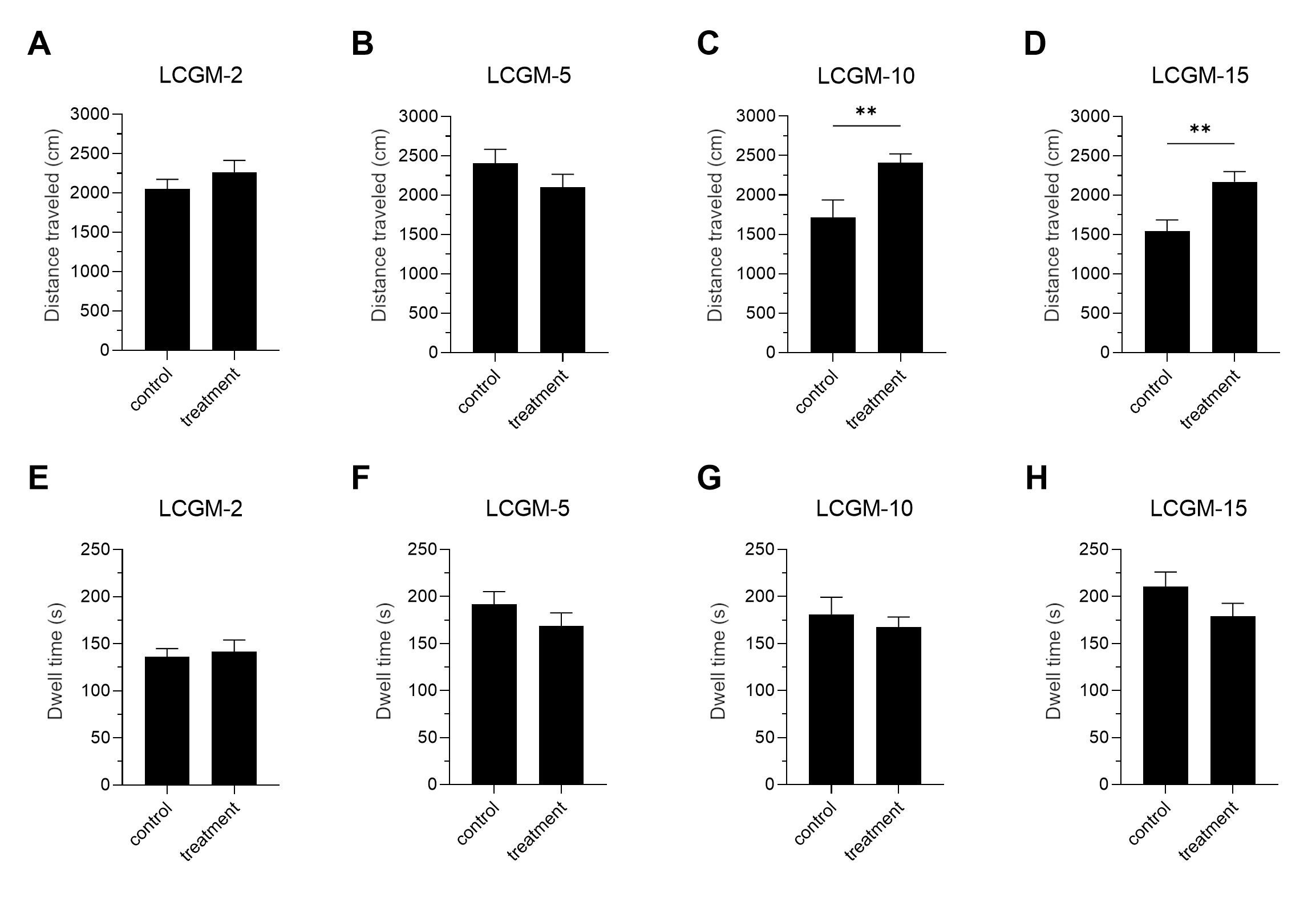


**Figure S3**. **The behavioral effects of LCGM peptides treatment in *Danio rerio* in the NTT.** Peptides LCGM-2, LCGM-5, LCGM-10 and LCGM-15 were tested for in vivo activity in zebrafish after a single i.p. injection at a dose of 1 mg/kg. (**A-D**) Distance traveled (in cm) by fish was significantly increased after LCGM-10 and -15 administration. (**E-H**) The bottom-dwelling duration (in seconds) was not affected by any treatment. The results are presented as the mean and SEM. **- p<0.01 vs corresponding control group; two-way ANOVA followed by two-stage linear step-up procedure of Benjamini, Krieger and Yekutieli, at a q threshold of 0.05. (N=16 in each treatment and control group). For statistics see Table 2.


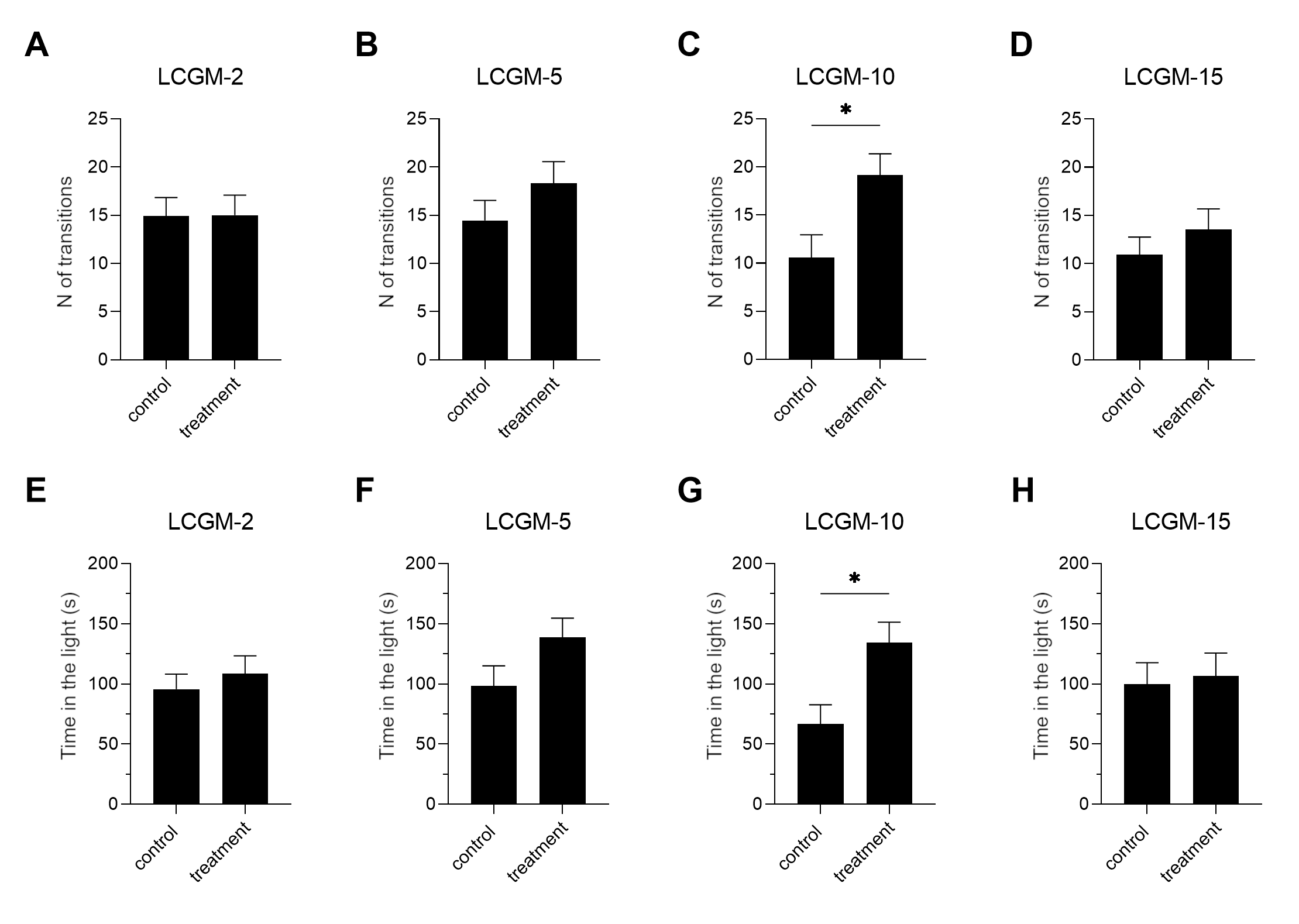


**Figure S4.** **The behavioral effects of LCGM peptides treatment in *Danio rerio* in the LDB test.** Peptides LCGM-2, LCGM-5, LCGM-10 and LCGM-15 were tested for in vivo activity in zebrafish after a single i.p. injection at a dose of 1 mg/kg. (**A-D**) The number of dark-light transitions and (**E-F**) the time spent in the light (in seconds) were increased when fish received LCGM-10 peptide. The results are presented as the mean and SEM. *-p<0.05 vs corresponding control group; two-way ANOVA followed by two-stage linear step-up procedure of Benjamini, Krieger and Yekutieli, at a q threshold of 0.05. (N=16 in each treatment and control group). For statistics see Table 2.


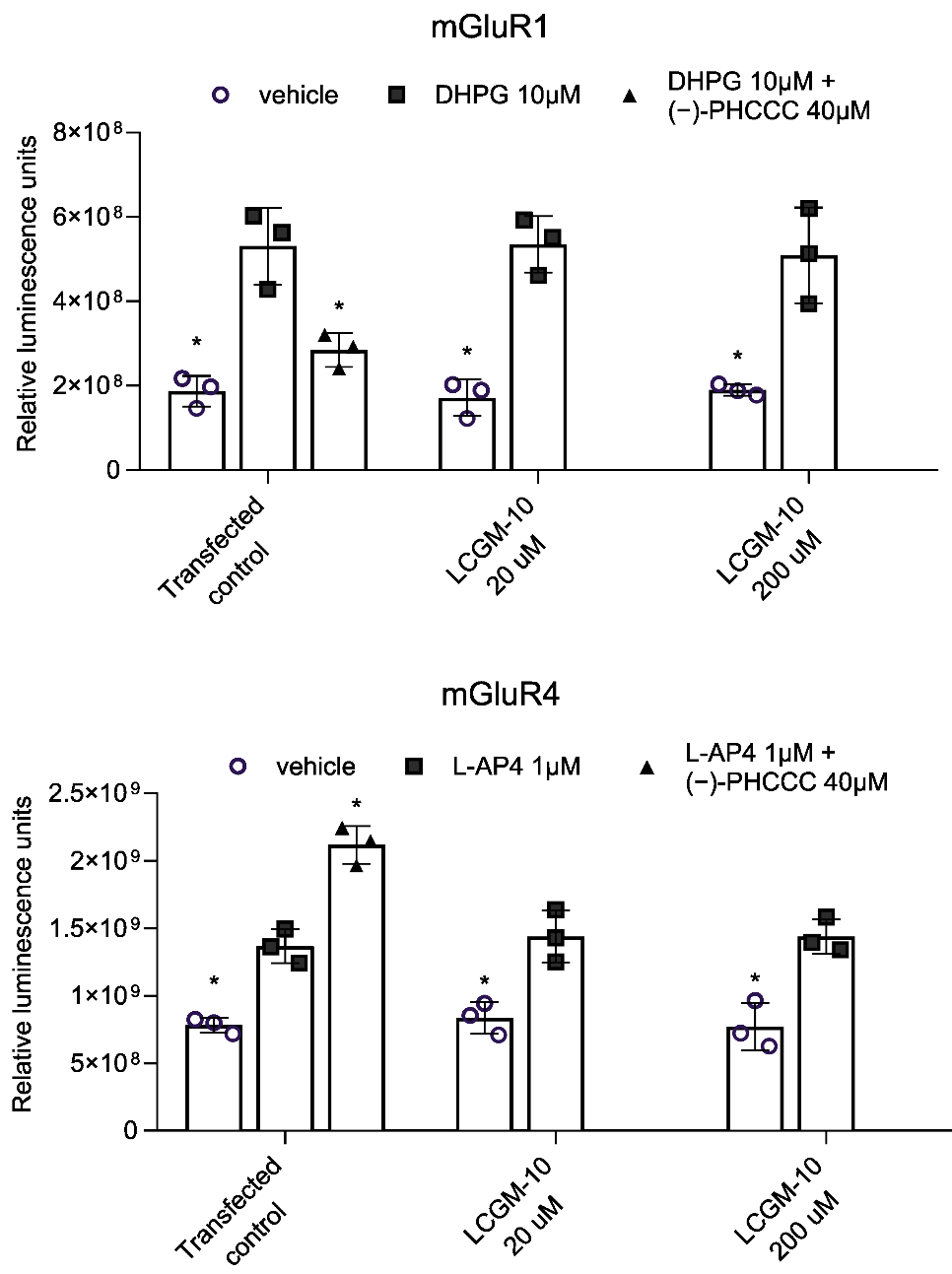


**Figure S5. The mGluR1- and mGliR4-luciferase reporter assay results.** HEK293 cells transfected with genetic reporter systems were treated with DHPG (10 µM) or DHPG (10 µM) + (−)-PHCCC (40 µM) in the case of mGluR1 and with L-AP4 (1 µM) or L-AP4 (1 µM) + (−)-PHCCC (40 µM) in the case of mGluR4. LCGM-10 was administered at a dose of 20 and 200 µM. Introduction of mGluR1 and mGluR4 agonists induced luciferase signals indicating the receptors’ activation. (−)-PHCCC inhibited DHPG-induced mGluR1 activity and enhanced L-AP4-induced mGluR4 activity, corresponding to its NAM and PAM functions respectively. LCGM-10 did not influence the basal activity of the reporters and the activation produced by the agonists in both assays. The data are presented as the mean ± standard deviation for three biological replicates (N=3 in each group). * P < 0.01 versus the transfected control + control agonist.


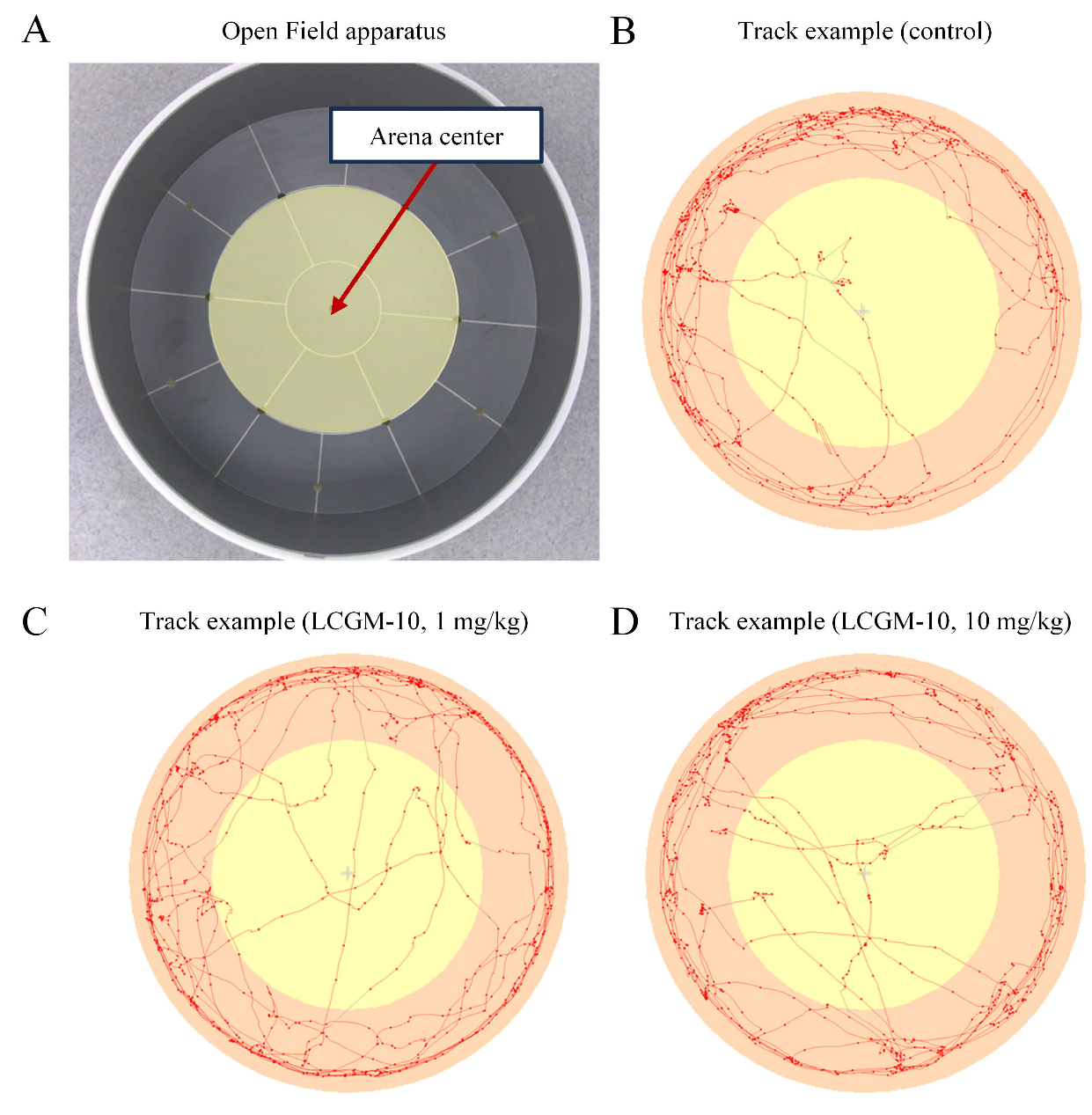


**Figure S6**. **Open field test.** General view of the arena (**A**). **Animal track visualization.** Examples of tracks: Control (B); LCGM-10, 1 mg/kg (C); LCGM-10, 10 mg/kg (D)


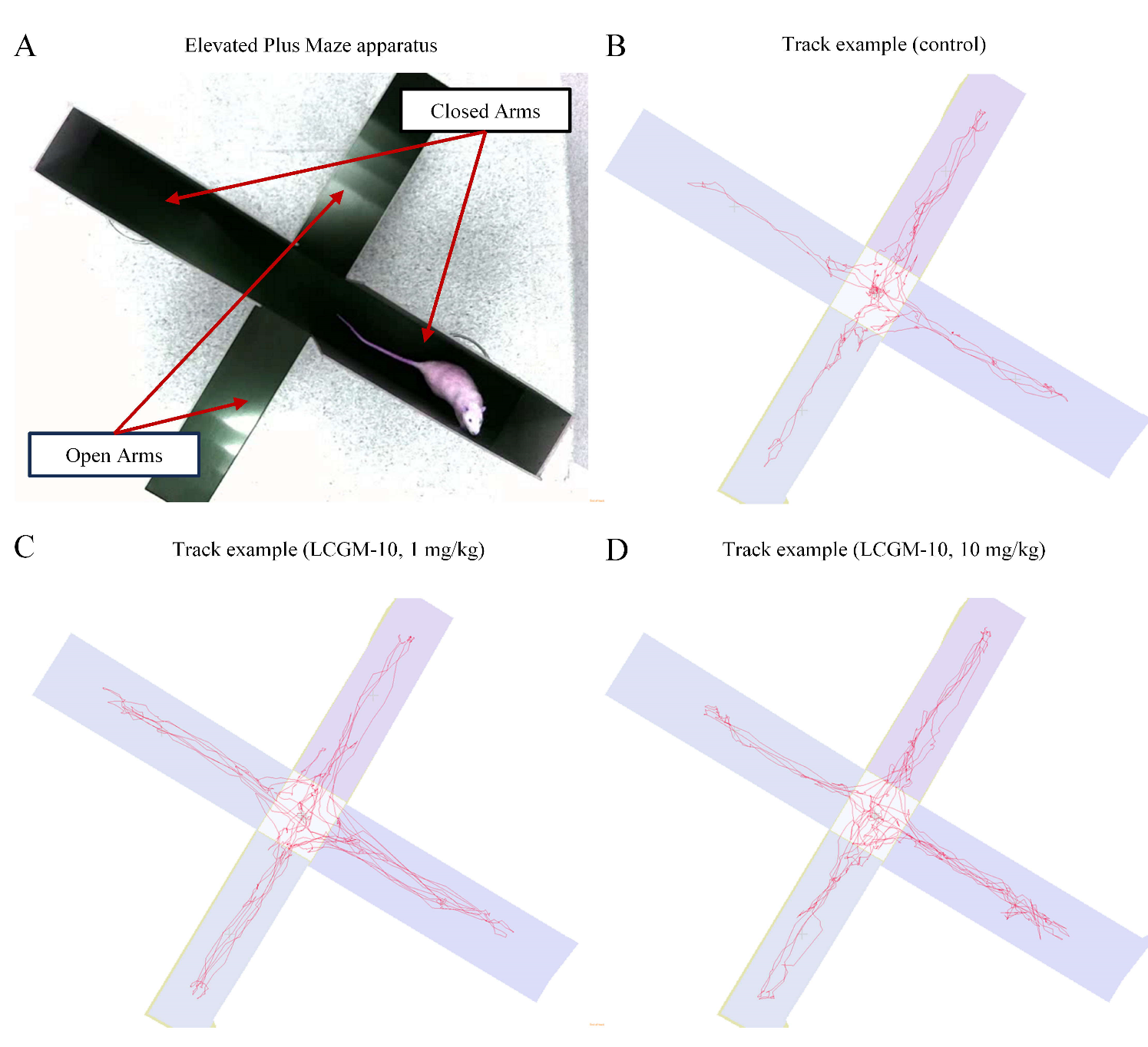


**Figure S7**. **Elevated plus maze test.** General view of the arena (**A**). **Animal track visualization.** Examples of tracks: Control (B); LCGM-10, 1 mg/kg (C); LCGM-10, 10 mg/kg (D)

**Table S1**. OF test results

| **Treatment** | **Control** | **LCGM-10,**  **1 mg/kg** | **LCGM-10,**  **10 mg/kg** | **One-way ANOVA** |
| --- | --- | --- | --- | --- |
| Distance traveled in the center (cm) | 176.6±42.8 | 180.1±30.8 | 195±34.6 | F_2, 27_ = 0.07,  P = 0.9 |
| Distance traveled near walls (cm) | 2972.5±109.4 | 3740.0±132.9* | 3573.9±178.8* | F_2, 27_ = 7.9,  P = 0.002 |

Data presented as mean ±SEM. * - p<0.05 vs control group *post hoc* Holm–Šídák test.

**Table S2**. **Rats body weight during the delay discounting experiment**

| **Day of the experiment** | **1** | **15** | **30** | **33** | **45** |
| --- | --- | --- | --- | --- | --- |
| **Animals’ identification №** | **Day 1 of the operant training** | **Day 15 of the operant training** | **Day 30 of the operant training** | **Before single LCGM-10 administration** | **Before chronic LCGM-10 administration** |
| 1-9 | 398 | 348 | 340 | 329 | 338 |
| 1-10 | 366 | 303 | 316 | 312 | 307 |
| 2-1 | 356 | 308 | 317 | 299 | 304 |
| 2-2 | 397 | 343 | 329 | 332 | 341 |
| 2-3 | 399 | 348 | 345 | 354 | 348 |
| 2-5 | 397 | 337 | 341 | 352 | 341 |
| 3-4 | 394 | 335 | 331 | 337 | 335 |
| 3-5 | 376 | 321 | 311 | 325 | 321 |
| 3-6 | 399 | 335 | 344 | 322 | 335 |
| 3-7 | 351 | 304 | 293 | 297 | 291 |
| 3-8 | 382 | 323 | 319 | 334 | 323 |
| 3-10 | 351 | 301 | 295 | 293 | 297 |
| 3-11 | 405 | 345 | 352 | 344 | 355 |
| 4-3 | 381 | 330 | 324 | 327 | 331 |
| 4-8 | 357 | 303 | 300 | 308 | 298 |
| 4-10 | 362 | 308 | 314 | 320 | 312 |
| 5-2 | 347 | 287 | 299 | 298 | 287 |
| 5-3 | 351 | 303 | 305 | 296 | 291 |
| 5-4 | 381 | 321 | 315 | 331 | 321 |
| 5-5 | 371 | 313 | 311 | 317 | 323 |
| 5-7 | 386 | 328 | 316 | 335 | 328 |
| 5-9 | 372 | 310 | 314 | 317 | 310 |
| 5-10 | 384 | 332 | 337 | 328 | 333 |
| 6-3 | 353 | 302 | 308 | 310 | 297 |
| 6-4 | 373 | 310 | 324 | 321 | 317 |
| 6-5 | 402 | 351 | 338 | 344 | 338 |
| 6-7 | 371 | 312 | 322 | 320 | 312 |
| 6-8 | 362 | 309 | 292 | 314 | 319 |
| 6-10 | 387 | 326 | 337 | 329 | 336 |
| 6-11 | 393 | 342 | 330 | 325 | 336 |

Values are presented for the 30 HI animals used in the main testing.
